# Ultrasound Imaging by Thermally Tunable Phononic Crystal Lens

**DOI:** 10.3390/ijms22157966

**Published:** 2021-07-26

**Authors:** Yuqi Jin, Arup Neogi

**Affiliations:** 1 Department of Physics, University of North Texas, P.O. Box 311427, Denton, TX 76203, USA; yuqijin@my.unt.edu; 2 Department of Mechanical Engineering, University of North Texas, 3940 North Elm Suite F101, Denton, TX 76207, USA; 3 Center for Agile and Adaptive Additive Manufacturing, 3940 North Elm Suite, Denton, TX 76207, USA

**Keywords:** tunable lens, ultrasonic detection, deep detection, phononic crystal, hydrogel

## Abstract

This work demonstrates the detections and mappings of a solid object using a thermally tunable solid-state phononic crystal lens at low frequency for potential use in future long-distance detection. The phononic crystal lens is infiltrated with a polyvinyl alcohol-based poly n-isopropyl acrylamide (PVA-PNIPAm) bulk hydrogel polymer. The hydrogel undergoes a volumetric phase transition due to a temperature change leading to a temperature-dependent sound velocity and density. The temperature variation from 20 °C to 39 °C changes the focal length of the tunable solid-state lens by 1 cm in the axial direction. This thermo-reversible tunable focal length lens was used in a monostatic setup for one- and two-dimensional mapping scans in both frequency domain echo-intensity and temporal domain time-of-flight modes. The experimental results illustrated 1.03 ± 0.15λ and 2.35 ± 0.28λ on the lateral and axial minimum detectable object size. The experiments using the tunable lens demonstrate the capability to detect objects by changing the temperature in water without translating an object, source, or detector. The time-of-flight mode modality using the tunable solid-state phononic lens increases the signal-to-noise ratio compared to a conventional phononic crystal lens.

## 1. Introduction

Phononic crystals are artificially engineered crystals with a periodic arrangement [1,2,3,4] of scatterers in an ambient medium. Based on incident wavelength, these phononic crystals can either cause Bragg scattering [5,6] or behave as a homogeneous material [7,8]. As in crystals with electronic bandgap, the transient behavior of phononic crystals can be modified by changing the lattice diameter, spacing, or arrangement as needed for an application. The flexibility of these phononic crystals has led to the design of new classes of lenses [6], filters [9], beam splitters [10], and decomposition devices [11]. A phononic crystal lens has been demonstrated to behave similarly to a homogeneous optical lens beyond the long-wavelength limit [12]. Phononic crystal-based lenses can also yield sub-wavelength resolution in the evanescent near-field by negative refraction and by breaking the diffraction limit using the meta-materials properties [6,13,14,15,16]. However, these artificially designed lenses have barely been used to image any real object for practical application. Moreover, these lenses are passive structures, and the operating wavelength is fixed for a designed structure.

There are three main categories of nondestructive imaging or mapping modality grouped in terms of the measurement techniques: echo-intensity [17,18], time-of-flight [19,20,21], and elastography [22,23]. Each of these techniques modalities has its advantages. The time-of-flight mode imaging provides temporal information about the size and the location of an object in an ambient medium [20]. Echo-intensity detection can distinguish the contrast between an object and ambient medium in terms of acoustic impedance [18] and is normally presented in the frequency domain. For a standardized ambient media, echo-intensity detection can also provide information about the location of the object in the axial direction based on the overall attenuation from the ambient material. Elastography is a technique that differentiates the object’s elasticity contrast from its ambient environment using active [24] or passive [25,26,27] experimental methods.

Poly-vinyl alcohol-based poly n-isopropyl acrylamide (PVA-PNIPAm) hydrogel is a polymer that undergoes a volumetric phase transition at ~32–33 °C. The elasticity of the polymer changes due to the phase transition and can result in over a 10% change in the velocity of sound in the PVA-PNIPAm [23]. This variance in sound velocity can be used to thermally tune the effective mechanical properties of the phononic lens interstitially filled with PVA-PNIPAm, and consequently achieve a tunable focal length lens.

**Figure 1:**
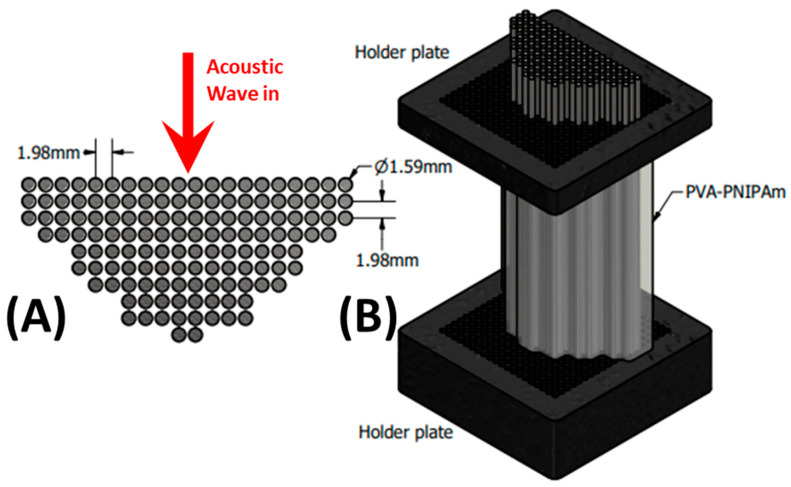
Design of the tunable solid-state lens. (**A**) The configuration of stainless-steel rods array as the scatterers in the phononic crystal structure. (**B**) The configuration of the lens including holder plates of the scatterer array and interstitially filled with thermally tunable hydrogel.

**Figure 2:**
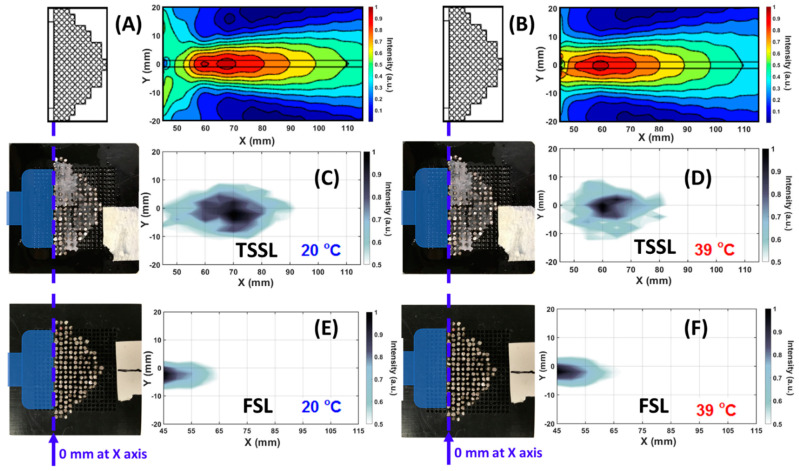
Sound intensity maps of TSSL (infilled with hydrogel) comparing with FSL (infilled with ambient water). Origin point of the *X*-axis in the contours was at the interface between transducer and lens. (**A**,**B**) illustrated numerical simulation of the focusing fields of TSSL at 20 °C and 39 °C. (**C**) Focusing point of TSSL at 20 ℃. (**D**) Focusing point of TSSL at 39 °C. (**E**) Focusing point of FSL at 20 °C. (**F**) Focusing point of FSL at 39 °C.

**Figure 3:**
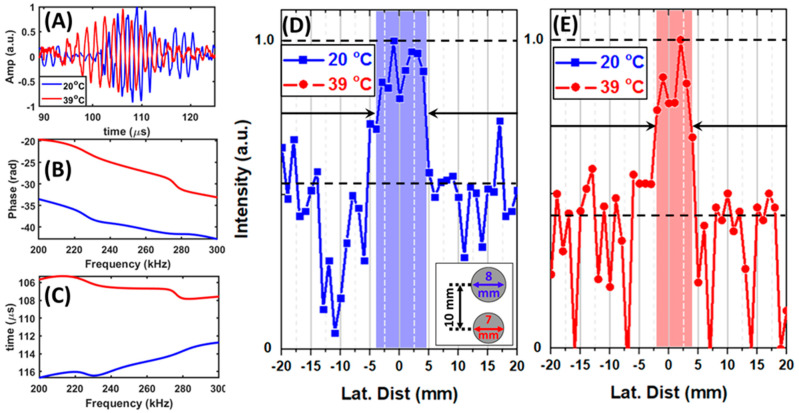
One-dimensional frequency-domain echo-intensity mode detection of two 3D printed ABS plastic rods in ambient water. The rods were located along the *Y*- (lateral) axis (Figure 2). The 7 mm diameter rod was 60 mm away from the transducer surface. The 8 mm diameter rod was 70 mm away from the transducer. (**A**) Temporal reflection waves detected from the target rods using TSSL at 20 °C and 39 °C. (**B**) The monochromatic components’ phase delay in the range from 0.2 MHz to 0.3 MHz obtained by FFT of the signal showed in (**A**). (**C**) The arrival time of the monochromatic components in the range from 0.2 MHz to 0.3 MHz from the phase information illustrated in (**B**). The results for the one-dimensional echo-intensity mode detection was obtained by FFT of the temporal waves in (**A**) shown by the blue solid line with squares (TSSL at 20 °C) in (**D**) and red dash-dot line with circles (TSSL at 39 °C) in (**E**).

**Figure 4:**
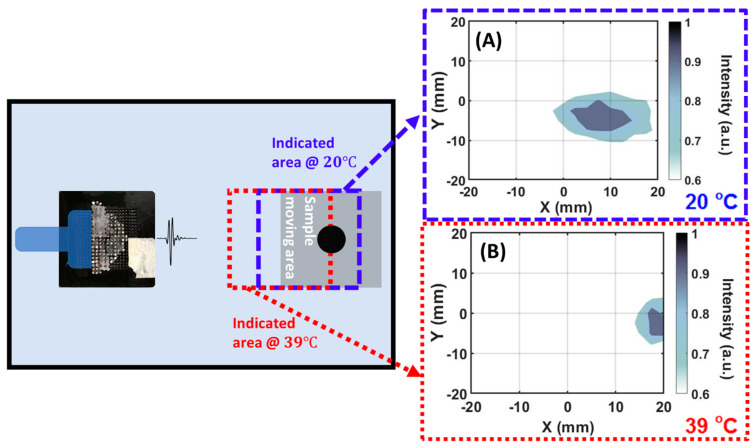
2D frequency domain echo-intensity mode mappings by TSSL at 20 °C and 39 °C. A 10 mm diameter aluminum rod moved on the *X*- and *Y*-axis in a 40 mm by 40 mm area with a 2 mm interval on both axes. TSSL scanned data recorded at 20 °C (**A**) and 39 °C (**B**) with the sample moving in the same area.

**Figure 5:**
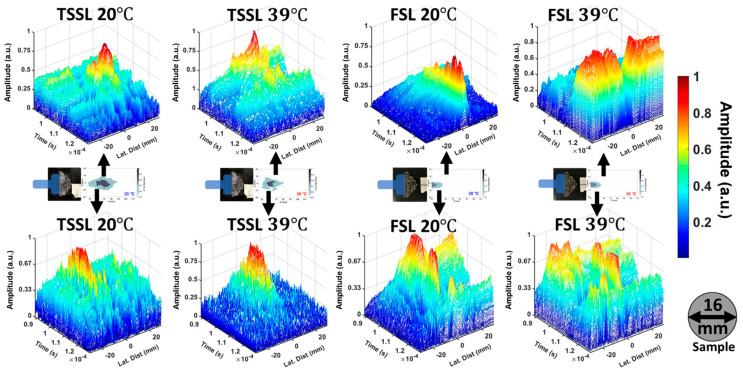
One-dimensional temporal domain time-of-flight mapping. A 16 mm diameter Aluminum rod translated on lateral direction (*Y*-axis) by a distance of 60 mm with 1 mm interval. The translating axis on *X*-axis was located 70 mm away from the transducer surface (Upper four subfigures (**A**,**B**,**E**,**F**)) and 60 mm away from the transducer surface (Lower four subfigures (**C**,**D**,**G**,**H**)).

In this study, the thermally tunable solid-state phononic crystal lens (TSSL) (Figure 1) is utilized for both frequency domain echo-intensity and temporal domain time-of-flight based one- or two-dimensional scans in a monostatic setup. As the temperature of the lens is gradually raised from room temperature (20 °C), the focal length of the lens shifts from 10.29λ to 8.82λ from the transducer surface (Figure 2). This all-acoustic technique is utilized without any advanced signal processing technique and can laterally resolve 1.03 ± 0.15λ. in one/two-dimensional echo-intensity mode (Figure 3 and Figure 4) and 2.35 ± 0.28λ. in the axial direction using time-of-flight mode (Figure 5). Compared with a water-based focusing sonic lens (FSL), the polymer-based TSSL demonstrates its tunability in its focal length and far-field detection capability, which was characterized and explained in our previous work [28]. In this work, detections and mappings with the tunable solid-state lens are demonstrated, and the practical potential of the lens is investigated through the echo-intensity and time-of-flight measurements. The lateral and axial minimum detectable object size of the tunable solid-state lens was also experimentally characterized in hard material target samples.

## 2. Results and Discussion

Focusing for the lens configurations both with and without hydrogel are shown in the intensity maps of Figure 2 at the operating frequency of 0.2 MHz, well within the homogenization zone as shown in the experimental section. For lens characterization, the full width at half the maximum of the transmitted signal is used for analysis. Objects are placed at the focal length of the lens and images are reconstructed from pulse-echo data. We observed from Figure 2E,F that the focal points of the water-based FSL are located at 45 mm away from the surface of the transducer. This position was located about 25 mm away from the apex of the phononic crystal lens. The FSL intensity maps show no appreciable effect of temperature on the lens’s focal length as the water temperature is raised from 20 °C to 39 °C. The polymer’s infiltration in the lens (TSSL) leads to an increase in the focal length of the lens to about 70 mm at room temperature (Figure 2A,C). Due to the change in the speed of sound and effective density of the polymer-filled phononic crystal, the lens’s focal length increases. Figure 2B,D show that as the temperature is increased to 39 °C, the focal length of the TSSL lens reduces to about 60 mm in the axial direction. The numerical results (A and B) were qualitatively agreed with experimental results (C and D). The focal point of the lens at 39 °C is comparatively sharper than at 20 °C. The width of the focal point observed at 20 °C was clearly larger than the focal width at 39 °C. The enlarged focal point was expected to induce lower spatial resolution in detection.

The focalization mechanism illustrated in the Appendix A. The physical mechanism of focusing the ultrasonic was achieved differently from the acoustic meta-lens, which normally has a negative refraction index [29].

The temperature-dependent change in the lens’s focal length property has been used to detect actual objects submerged in water. To test the detection feasibility of TSSL, two cylindrical acrylonitrile butadiene styrene (ABS) plastic rods with a diameter of 8 mm and 7 mm were 3D printed to test the lenses’ detection capability. The structure is shown in the inset of Figure 3. The cylindrical rods were separated by a center-to-center distance of 10 mm, which corresponds to the tunable range of the TSSL (Figure 2C,D). These structures were placed along the lens’s axial direction at the distance of 60 mm and 70 mm from the transducer surface as the blue dash lines indicated in Figure 2, which the one-dimensional scan along the Y direction. The 7-mm rod was placed closer to the TSSL than the 8 mm rod. Figure 3A illustrated the original reflected broadband pulse envelopes recorded at 0 mm from the 1D scan. 

To determine the detailed temporal delay of the single-frequency component at 0.2 MHz, the wave envelopes were transformed to the frequency domain by FFT. The obtained phase of the frequency components in the range between 0.2 MHz and 0.3 MHz were illustrated in subfigure (B). In (C), the phase delay was translated in arriving time for a better illustration. The figures showed that the object detected at 39 °C was physical closer than the object found at 20 °C respected by the transducer. For clear estimations on the width difference between the two detected objects, the frequency domain single frequency component information at 0.2 MHz was sorted by the scanned direction along the lateral axis. Figure 3D,E show the transmitted ultrasonic wave’s spatial profile through the lens at 20 °C and 39 °C. The wavelength of the emitting transducer used for the detection was about 6.8 mm. Our experimental observation showed that the TSSL detected the 8 mm diameter rod at 20 °C and the 7-mm diameter rod (located closer to the lens) at 39 °C without any axial translation of either the sample or the lens. Estimating the scanned object width values were from the intensity profiles with the averaged amplitude level between maximum and averaged noise. The object width accuracy of the TSSL operated in the 1D echo-intensity mode at 20 °C is 7% less than that at 39 °C. The loss of accuracy is due to the larger spatial dispersion of the TSSL at room temperature, as observed from Figure 2C,D.

Figure 4 shows the result of two-dimensional monostatic echo-intensity mode mapping of a 10 mm diameter aluminum rod in water ambient. In the experiments, the rod (sample) was translated within a 40 mm × 40 mm square area with a 2-mm interval on both the X- and Y-axes with the position of the source transducer and TSSL fixed. The center of the detection area was 80 mm away from the transducer surface on the axial (X) axis. Figure 4A,B show the reflection intensity profiles at 0.2 MHz of the object scanned by TSSL at 20 °C and 39 °C within identical scanning cross-section of the sample with motion. An increase in the temperature from 20 °C to 39 °C results in a scanned area shift backward by 10 mm on the X- (axial) axis. Both subfigures A and B showed an 11-mm wide object. Figure 4A depicts an ellipsoidal rod due to the focused beam’s dispersion in the axial direction at 20 °C (Figure 2A).

**Figure 6:**
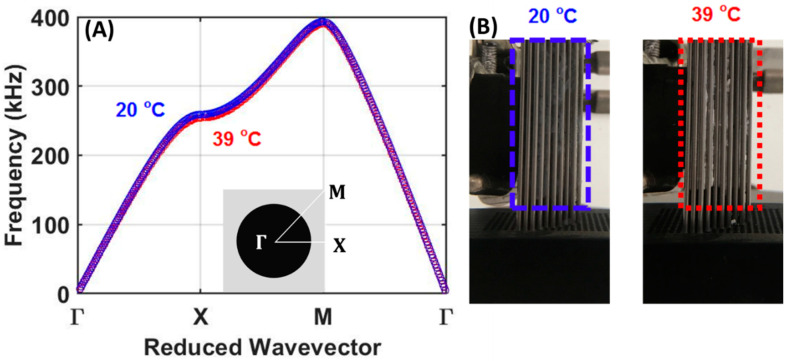
(**A**) Band structure of the first band of the PVA-PNIPAm filled phononic crystal at 20 °C (blue) and 39 °C (red) using the measured hydrogel density and speed of sound. Γ, Χ, and Μ points refer to the location of (0,0), (0,1) and (1,1) in the unit reciprocal unit cell. (**B**) Photographs of the TSSL at 20 °C and 39 °C ambient temperatures.

**Figure A1:**
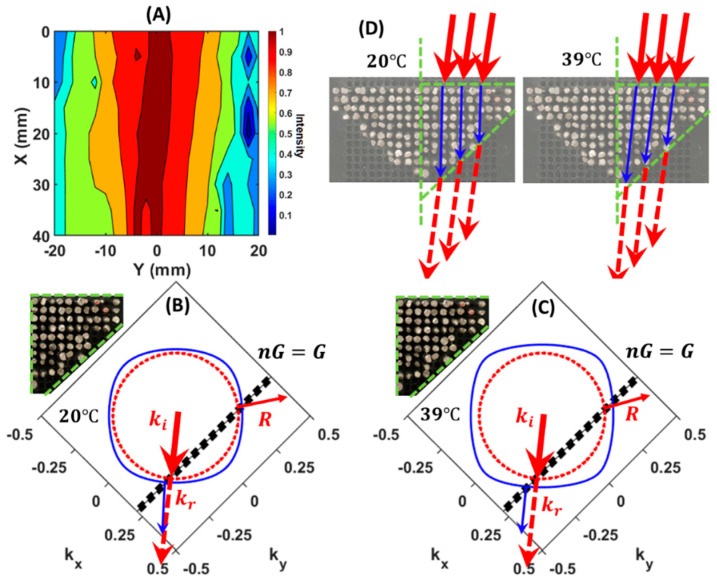
(**A**) Sound intensity field of the source planar transducer. The value of 0 mm on the *Y*-axis refers to the surface of the transducer. (**B**) Equi-frequency contour (EFC) of TSSL at 20 °C. (**C**) EFC of TSSL at 39 °C. (**D**) Illustration of the focusing mechanism based on the refracted wave vectors from (**B**,**C**) The unit of k_x_, k_y_ and G was 2π/a. The red arrows referred the wave direction in water. The blue arrows referred the wave direction in lens.

Figure 5 shows a comparison of the far-field monostatic 1D time-of-flight mode mappings using the TSSL and the same structure without the PVA-PNIPAm polymer (an FSL). This modality uses the time of flight measurement of the reflected waves from the object. The results are presented in 3D surface plots (Figure 6) and 2D color contours (Figure A1) in terms of the lateral distance (*Y*-axis), the time-of-flight of the reflected wave with the normalized linear intensity scale. The scanned sample was a 16 mm diameter aluminum rod translated along the Y direction (laterally) by 60 mm in steps of 1-mm intervals. In Figure 5, the top four plots were obtained from the experiments when the sample was about 70 mm (farther) away from the transducer’s surface on the *X*-axis. The bottom four plots were from the experiments when the sample was about 60 mm away from the transducer axially. Both TSSL and FSL were used for the temporal domain time-of-flight mode mapping under identical experimental conditions for comparison. The time-of-flight mode mapping was performed by lateral sweeping around the focal distance to detect the target object. The higher signal-to-noise ratio for the ultrasonic wave around the axial direction indicates the presence of an object. By detecting the temporal location and the reflected signal’s length, the depth and axial size of the object could be calculated. These reflected signals are compared to a standard reference with a well-calibrated speed of sound and acoustic properties. 

**Figure A2:**
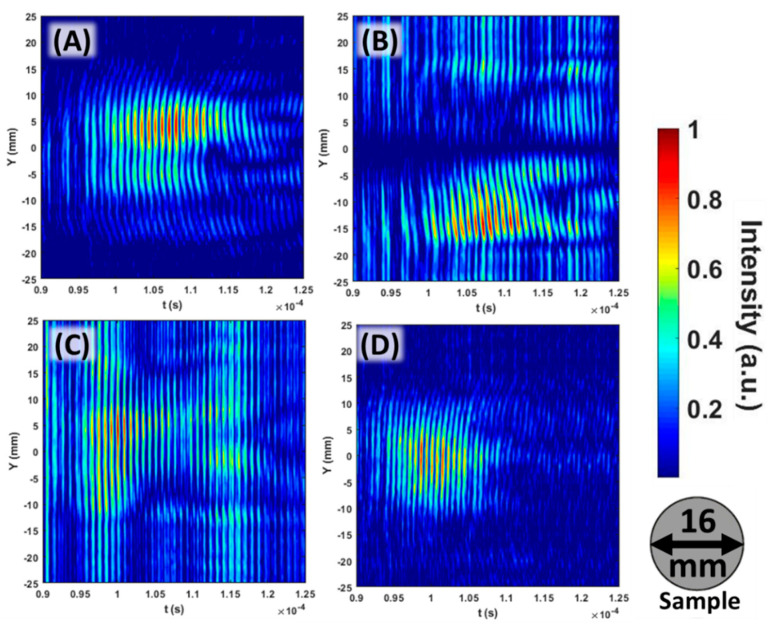
Two-dimensional contour version of the time-of-flight mode detection (Figure 5A–D) using TSSL at near and far distance at 20 °C and 39 °C. (**A**). Far distance (70 mm) between transducer and sample scanned by TSSL at 20 °C. (**B**). Far distance (70 mm) between transducer and sample scanned by TSSL at 39 °C. (**C**). Near distance (60 mm) between transducer and sample scanned by TSSL at 20 °C. (**D**). Near distance (60 mm) between transducer and sample scanned by TSSL at 39 °C.

Figure 5 shows that the TSSL exhibits an improved signal-to-noise ratio when the object is at 70 mm for a lens temperature of 20 °C. At 39 °C, the object at 60 mm exhibits a higher signal-to-noise ratio. In order to have a better view of the contrast in signal-to-noise ratio under on-focusing and off-focusing conditions, the results were presented in 3D plots to show the amplitude difference. The conventional 2D contours versions of the TSSL results were posted in the Appendix A Figure A2.

From Figure 5A,D, the center of the reflected signal at 20 °C is observed at 107.1 µs and 93.8 µs (39 °C) from the sample to the TSSL which corresponds to the distances between the transducer surface and samples. The axial dimension of the scanned aluminum rod could be obtained from the half maximum of the temporal length of the reflection from the signals at 20 °C (farther sample) and 39 °C (closer sample), which were 7.27 µs and 5.52 µs respectively. As the sound speed in aluminum is 6400 m/s, the axial estimated values of the scanned sample were 23.2 mm and 17.6 mm.

The thermal sensitivity of the tunability of TSSL has been demonstrated in the above results. By changing the polymer phase to above the volume phase transition temperature, the focal length could be reversibly changed from 10.29λ to 8.82λ away from the transducer’s surface. From the monostatic 1D frequency-domain echo-intensity mode detection, the lateral minimum detectable object size of the PVA-PNIPAm infilled TSSL was illustrated as detecting a 1.03λ wide object with a 0.15λ error. Since the operating frequency of the TSSL was in its first transmission band, a sub-wavelength minimum detectable object size was not expected. The lateral minimum detectable object size (1.03λ) of the TSSL was not compromised by the dispersion of the acoustic waves in the polymer and was close to the operating wavelength (6.8 mm). The axial minimum detectable object size of the TSSL that provides information about the rod’s diameter was observed to be 2.35λ.

Compared to a tunable solid-state lens with a similar shape focusing on the sonic lens, the tunable solid-state lens focused the sound in the far-field which is more suitable for practical detections. Biomedical ultrasound imaging and detection are mostly operating in high frequency, such as 10 MHz for the smaller lateral minimum detectable object size. Higher frequency introduces larger attenuation and dispersion effects, reducing detection depth and penetration. In this study, a low operating frequency (0.2 MHz) tunable solid-state lens detections and mappings demonstrated about one wavelength lateral minimum detectable object size, which showed the potential possibility of clear, long-distance detection.

Further study on this novel type of phononic crystal lens can focus on increasing the lens’s tunability and increasing the scanning speed between the variable focal points. The use of the different types of phase transition hydrogel [30], even microgel [31], or viscoelastic polymer with larger modulation of its physical properties, can be applied in TSSL to obtain a larger tunable focusing distance. Instead of conventional heating of the TSSL, electromagnetic waves such as infrared radiation [9] and radio-frequency electromagnetic field [32] can be a viable option.

## 3. Material and Methods

The phononic crystal structure used in this study was designed and fabricated to have a 50.1% filling fraction of stainless-steel scatterers in water (FSL) or PVA-PNIPAm (TSSL) in the square lattice configuration. The diameter of the stainless-steel rods was 1/16-inch (1.59 mm) with a lattice constant of 5/64-inch (1.98 mm) periodic arrangement. Based on our previously reported temperature-dependent density and sound speed in PVA-PNIPAm hydrogel [33], the temperature dependence of the dispersion relation of the first transmission band for the hydrogel infilled phononic crystal was calculated and presented in Figure 6A. The actual phononic crystal structure infiltrated with hydrogel before and after phase transition is shown in Figure 6B. The stainless-steel rod has the elastic modulus 195 GPa, Poisson’s ratio 0.29, and density 7500 kg/m^3^ from the datasheet. The dynamic bulk modulus of the PVA-PNIPAm hydrogel was calculated as a product of density (ρ) and square of speed of sound ($K=\rho c^{2}$), which was 1.65 GPa at room temperature and 2.42 GPa at 39 °C [34]. The effective sound velocity of the hydrogel infilled PnC lens is 2150 m/s at room temperature and 2289 m/s at 39 °C.

The numerically simulated results of sound intensity fields illustrated in Figure 2A,B were performed by finite element analysis (FEA) based COMSOL Multiphysics software using pressure acoustic and solid mechanics module. The acoustic transducer was defined as a plane wave source. The properties of ambient water were selected from the COMSOL library with the speed of sound of 1480 m/s at 295 K ambient, and density of 998 kg/m^3^. The Young’s modulus, speed of sound, and a density of stainless-steel rods were set to 200 GPa, 5800 m/s, and 7850 kg/m^3^ respectively. The speed of sound and density values of PVA-PNIPAm hydrogel were 1345.4 m/s and 1048.9 kg/m^3^ at 20 °C 1425.5 m/s 1269.3 kg/m^3^ at 39 ℃ which were experimental measured in our previous works [33,34].

**Figure 7:**
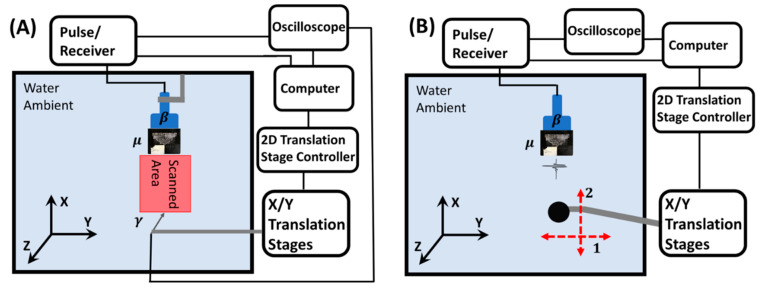
(**A**) Experimental setup of sound pressure field raster scan. (**B**) Experimental setup of monostatic frequency echo-intensity and time domain time-of-flight modes mappings in both 1D and 2D. One-dimensional detection was performed by moving the sample along arrow 1 (*X*-axis). 2D mapping was achieved by translating the object in both arrow 1 and 2 (*X*- and *Y*-axis). In the illustrations, β was transducer. µ indicated lens. Arrow γ represented needle hydrophone.

Figure 7A shows the bistatic experiment setup of sound intensity raster focusing point maps (Figure 2). A one-inch (25.4 mm) Olympus diameter planar transducer V301 and the lens in a deionized water tank were fully emerged and aligned for the transmission measurements. The transducer-generated broadband pulses at 0.5 MHz center frequency with effective bandwidth from 0.1 MHz to 0.9 MHz at 500 Hz repetition rate were focused using the lens. The 0.5 MHz center frequency immersion transducer was used as the acoustic source. Our target frequency of 0.2 MHz in frequency domain experiments was located close to the Gaussian-liked distribution bandwidth edge from 0.1 MHz to 0.9 MHz, which led to a relatively low initial signal-to-noise ratio. 

Other energy loss sources also exist in the system, such as impedance mismatch between ambient water and TSSL and the hydrogel’s attenuation. A needle hydrophone (Müller-Platte) with a tip diameter of 1 mm was used to measure the outcoming signal from the lens. The tip was used to map the acoustic pressure within a 40 mm (Y) by 70 mm (X) area with a 2-mm interval along the *Y*-axis and a 5-mm interval along the *X*-axis. The needle was raster-scanned using a custom MATLAB code and controlled by the computer. The program-controlled the Newmark NSG-G2-X2 translation stage to linearly translate with a step size of 0.5 mm/s and a time delay of 15 s to facilitate data accumulation. It also controlled the two-dimensional Newmark LC-200-11 linear translation stage holding the needle hydrophone. The temporal signal from the hydrophone was averaged (512 time windows) and acquired in the time domain channel on a Tektronix MDO 3024 oscilloscope. In the frequency domain echo-intensity mode detection results, reflection intensity was described as a single frequency component at only 0.2 MHz. The monochromatic information at 0.2 MHz was obtained from a time domain to frequency domain conversion of the recorded time-domain reflection signal by fast Fourier transformation (FFT). 

With the single-frequency information at 0.2 MHz, the minimum detectable object size of the echo-intensity mode detection results (Figure 3 and Figure 4) can be analyzed based on its wavelength of 6.8 mm in ambient water. Figure 7B illustrates the monostatic setup of the experiments which produced results in Figure 3, Figure 4 and Figure 5. In the one-dimensional experiments, the sample was translated along arrow 1 (*Y*-axis) with 1-mm interval. In the two-dimensional experiment, the sample was translated in a 40 mm × 40 mm area along the direction of the arrows 1 and 2 (*Y*- and *X*-axis) with a 2-mm interval as indicated in Figure 7B.

## 4. Conclusions

This work demonstrated the first practical experimental study of a thermal tunable solid-state phononic crystal focusing lens prototype. The results demonstrated the feasibility of applying a hydrogel infilled tunable solid-state lens in frequency domain echo-intensity and temporal domain time-of-flight modes monostatic detections and mappings in simple experimental environments. Comparing with the same shape water-infilled FSL, the tunability of the focusing capability of the TSSL were remarkable advantages.

## Data Availability

Data available for requesting to the corresponding author.

## Appendix A

In Figure A1, we posted the focusing mechanism of the TSSL in more detail. As the experimental sound intensity field (subfigure A) illustrated, with a planar ultrasound transducer source (at 0 mm on *Y*-axis), the near field effect provided slight convergence wave vectors as the source to the TSSL. In subfigure (B) and (C), we showed the 0.2 MHz equi-frequency contours of TSSL at 20 °C and 39 °C. On the lens’s closer edge respected to the transducer, the wave was incident on the normal direction. Since the farther curve edge of the lens has an approximately 45° slope. After 45° clockwise rotation, 0^o^ downward incidence in the EFCs described the emission vector from a prefect collimated source. In EFCs (subfigures B and C), we used approximately normal incident angle in the 45° rotated reciprocal unit cell to represent the around 45° edge lens curvature. Due to the near-field effect, we used a 5° incident angle as the source vector from the transducer to the lens in water. The red dash circle was indicated to the EFC of the ambient water. The dash black lines were indicated the momentum-matched conditions of $k_{i}=\mathrm{nG}+k_{r}$, where $k_{i}$ and $k_{r}$ were the incidence vector from transducer in water and resultant vector from the lens in water. G was the reciprocal lattice vector. And n was the integral numbers. The dash black line was the momentum-matched condition when n equals 1. The $k_{x}$ axis location of the black dash line was from the projection of the incidence vector $k_{i}$. From (B) and (C), the EFCs predicted the refraction angle results vectors in water at the outcoming region which equals to the refraction angle incidence vector.

However, the energy propagation direction in the TSSL was different at 20 °C and 39 °C as the blue arrows in subfigure (B) and (C) showed. Vectors R were the backward propagation side beams. At 20 °C (39 °C), the group velocity inside the TSSL propagated more (less) comparable with 0° vertical direction. As the illustration in subfigure (D) showed, at 20 °C (39 °C), the smaller (larger) angle of internal refraction in TSSL resulted in a wider (narrower) outcoming converged beam and a farther (closer) focal length. In detail, at 20 °C, the source beam from the 1-inch diameter planar transducer had the beam radius around 11 mm and a slight convergence direction (near-field effect) at 10° as the red shadow and arrows illustrated. The wave propagated into the lens with the refracted angle around 2° respected to the horizontal direction which was obtained from the blue solid arrow in Figure A1B. The beam radius inside the lens was reduced to 10.1 mm at the curved lens edge around 13 mm on the axial axis respected to the edge close to the source transducer. The outcoming beam from the lens to ambient water had a refraction angle at 10° which was also showed in Figure A1B with the red dash arrow.

Based on the reduced beam radius (10.1 mm) and angle (10°), the calculated focal length was 70 mm as the following figure (A) showed. At 39 °C, the source beam from the transducer also had the radius around 11 mm and the slight convergence direction at 10° as the red shadow and arrows illustrated. The wave propagated into the lens with the refracted angle around 9°, which was obtained from the blue arrow in Figure A1C. Around the axial distance 15 mm apart from the flat edge of the lens, the beam radius reduced to around 7.9 mm at the curved lens edge. The outcoming beam from the lens to ambient water had a refraction angle at 10° which was also showed in Figure A1C. Based on the reduced beam radius (7.9 mm) and angle (10°), the calculated focal length was about 60 mm. The analytical EFC contours of the TSSL agreed with the behavior compared with the numerical and experimental determined temperature-dependent focal length in Figure 2.
